# Hyoid bone fusion and bone density across the lifespan: prediction of age and sex

**DOI:** 10.1007/s12024-016-9769-x

**Published:** 2016-04-25

**Authors:** Ellie Fisher, Diane Austin, Helen M. Werner, Ying Ji Chuang, Edward Bersu, Houri K. Vorperian

**Affiliations:** Vocal Tract Development Lab, Waisman Center, University of Wisconsin-Madison, 1500 Highland Ave, Rm 427, Madison, WI 53705-2280 USA; School of Medicine and Public Health, University of Wisconsin-Madison, 1300 University Avenue, Madison, WI 53706 USA

**Keywords:** Hyoid bone fusion, Bone density, Forensic science, In vivo, Computed tomography, 3D modeling

## Abstract

The hyoid bone supports the important functions of swallowing and speech. At birth, the hyoid bone consists of a central body and pairs of right and left lesser and greater cornua. Fusion of the greater cornua with the body normally occurs in adulthood, but may not occur at all in some individuals. The aim of this study was to quantify hyoid bone fusion across the lifespan, as well as assess developmental changes in hyoid bone density. Using a computed tomography imaging studies database, 136 hyoid bones (66 male, 70 female, ages 1-to-94) were examined. Fusion was ranked on each side and hyoid bones were classified into one of four fusion categories based on their bilateral ranks: bilateral distant non-fusion, bilateral non-fusion, partial or unilateral fusion, and bilateral fusion. Three-dimensional hyoid bone models were created and used to calculate bone density in Hounsfield units. Results showed a wide range of variability in the timing and degree of hyoid bone fusion, with a trend for bilateral non-fusion to decrease after age 20. Hyoid bone density was significantly lower in adult female scans than adult male scans and decreased with age in adulthood. In sex and age estimation models, bone density was a significant predictor of sex. Both fusion category and bone density were significant predictors of age group for adult females. This study provides a developmental baseline for understanding hyoid bone fusion and bone density in typically developing individuals. Findings have implications for the disciplines of forensics, anatomy, speech pathology, and anthropology.

## Introduction

The hyoid bone plays an important role in the functions of speaking, swallowing, prevention of regurgitation, and airway maintenance [1, 2]. Deviations in its position relative to the mandible or larynx, or in its angle of placement, can have significant ramifications for these important functions [2, 3]. The hyoid bone changes its position relative to the vertebrae during normal growth and development. In infancy, the hyoid bone lies just anteriorly to the second and third cervical vertebrae, and eventually lowers to the level of the fourth and fifth cervical vertebrae in adults [3]. Hyoid bone descent occurs concurrently with descent of functionally related structures, namely the larynx and epiglottis [4]. The only bone in the body that is free-floating, the hyoid bone maintains attachment with the mandible, tongue, styloid processes, thyroid cartilage, cricoid cartilage, clavicles, and sternum by ligament and muscle attachments [1]. Hyoid bone anatomy and physiology have been well documented due to the diverse nature of the muscle attachment sites as well as the multiple important functions that the hyoid bone supports. However, the precise nature and timing of hyoid bone fusion during the course of development has been less documented. A more thorough understanding of the timeline of fusion would elucidate the potential role of hyoid bone fusion in speaking and swallowing disorders and the effect of oral-pharyngeal functions on hyoid bone fusion, as well as helping improve precision in age-at-death and sex estimation.

The hyoid bone develops from the branchial arches and the midline mesenchymal condensation (MMC) between the second and third branchial arch cartilages. The hyoid body comes directly from the MMC, the lesser cornua and the stylohyoid ligament derive from the second branchial cartilages, and the greater cornua derive from the third branchial cartilages [4, 5]. The hyoid body and the two greater cornua contain pairs of ossification centers [5]. Ossification in the hyoid body is completed shortly after birth, but fusion in the other two pairs of ossification centers occurs later in life, if at all [1]. Some hyoid bones show a diarthrodial structure, a gap that resembles a synovial cavity in the body/cornua bone articulation. Kanetaka et al. [5] found that there is decreased prevalence of this structure with increasing age, suggesting that fusion occurs with aging when a diarthrodial structure is present early in life.

The majority of previous research on hyoid bone fusion comes from the forensic sciences. Only a handful of studies have used the entire developmental age range, and even fewer have examined in vivo hyoid bones from medical imaging studies of living individuals [6]. Findings are inconclusive on laterality (i.e., differences in fusion on right vs. left sides of the hyoid bone) and male versus female differences in hyoid bone fusion, possibly due to a lack of universal criteria for ranking fusion. For example, Parsons [7], Gupta et al. [8], Harjeet et al. [9], and Ito et al. [10] found no differences in laterality, while Shimizu et al. [11] found the right side more likely to fuse than the left. Similarly, Naimo et al. [12] and Miller et al. [13] found no male-versus-female differences in hyoid bone fusion, whereas Shimizu et al. [11] and O’Halloran et al. [1] found more instances of fusion in males than females. Gupta et al. [8] found fusion to occur nearly 5 years earlier in females, and Harjeet et al. [9] found female hyoid bones to have significantly more fusion than male hyoid bones after age 61. Of these studies, only three [9–11] define fusion and of those only one [9] provided images alongside their definition for increased clarity. In addition, only a handful of studies [2, 7, 12–14] have examined hyoid bone fusion of younger children and adolescents and as noted by Naimo et al. [12], most of these studies include fewer than 10 bones from children under the age of 18.

Interest in hyoid bone fusion in forensic science is related to age and sex estimation [1, 8, 9, 11–14] as well as to the role of fusion in fracture during perimortem strangulation or other trauma [15–18]. Models have been developed to estimate sex and age using size, volume, and shape measurements of the hyoid bone as well as the distance of the joint space between the greater cornua and hyoid body. An important and neglected variable is hyoid bone mineral density, particularly as it relates to age, sex, and fracture risk. Developmental trends and sex differences in bone density have been well documented in the radius, pelvis, and calcaneus bones [19–21]. However, to our knowledge, there are no published studies that address these trends specifically for the hyoid bone, nor are there published studies that address the relationship between hyoid bone density and its fusion, or whether density can be used in forensics to estimate age and sex. Thus, the relationship and interaction between fusion and bone density of the hyoid bone over the lifespan needs to be examined to determine if either of those factors can be used as a means of age and sex estimation.

The purpose of this study was to quantify the fusion of the hyoid bone across the entire lifespan, to assess developmental changes in bone density, and to determine if there is a relationship between bone density and fusion. Using a retrospective imaging database, intact hyoid bones from in vivo computed tomography (CT) scans, where the hyoid bone maintains an unaltered relation to nearby structures, were examined to determine the degree of fusion between the hyoid body and the right and left greater cornua. The distance between the hyoid body and the greater cornua was used to establish a hyoid fusion ranking system. This ranking system was used to document the varying degrees of fusion on each side of the hyoid bone in order to assess laterality, sex differences, and developmental trends in fusion. A three-dimensional model of each hyoid bone was generated using Analyze 11.0^®^ software to calculate the bone density in Hounsfield units (HU). Bone density was measured for each individual hyoid bone to contribute to the overall developmental understanding of hyoid bone form and function and more specifically to assess the relationship between bone density and fusion.

## Methods

### Subjects and scans

Using imaging studies performed at the University of Wisconsin Hospital and Clinics (UWHC), the Vocal Tract Development Laboratory has curated a large retrospective database of developmental head and neck CT scans to study the growth and development of vocal tract structures. All studies have received approval from the University of Wisconsin Health Sciences Institutional Review Board. For this study, 570 CT scans in DICOM format were visually inspected and determined to have the whole hyoid bone structure visible, with few enough artifacts for modeling to be possible. Out of the 570 scans across the lifespan, 136 scans met the additional criteria of equivalent number of males and females per age group as defined below, and imaging criteria of: (1) 2.5 mm slice thickness, (2) 14.0–22.0 cm FOV, (3) 512 × 512 matrix size, (4) reconstructed using a General Electric standard algorithm, and (5) first scan if an individual had repeated scans. These scans were then analyzed using image visualization and analysis software Analyze 11.0^®^ (AnalyzeDirect^®^; Overland Park, KS).

Scans were grouped into one of the following four age groups: pediatric: birth to 19 years (*n* = 46; 23 males, 23 females); young adult: ages 20–30 years (*n* = 30; 15 males, 15 females); middle-aged adult: ages 44–55 years (*n* = 28; 16 males, 12 females); and older adult: ages 75–95 years (*n* = 32; 12 males, 20 females).

### Three-dimensional hyoid bone models and bone density

Analyze 11.0^®^ software was used to open the DICOM CT scan files for visualization and three-dimensional (3D) modeling. Axial, sagittal, and coronal views were observed simultaneously to ensure accuracy while inspecting the degree of fusion and while creating the 3D model. Three-dimensional hyoid bone models were created with the Volume Edit tool in Analyze 11.0^®^ software with the standard algorithm in order to calculate bone density. The Volume Edit tool in Analyze allows for slice-by-slice tracing and stacking of the hyoid bone structure from CT images to create a 3D model. The volume threshold was manually adjusted by visual inspection on a per slice basis. Intensity in Hounsfield units (HU), commonly used to measure bone density, was then calculated from the outlined region defined by the 3D model generated using the Region of Interest tool (ROI) within Analyze 11.0^®^. See Cotter et al. [4] for additional detail on modeling methods. Lee et al. [22], Majumdar and Leslie [23], Schreiber et al. [24], and Lee et al. [25] all found that intensity in Hounsfield units calculated from CT scans is a suitable estimation for bone mineral density as calculated by dual-energy X-ray absorptiometry (DXA), which remains the gold standard for bone density measurement. In this paper, we will use intensity as an estimated measure of bone density.

### Fusion ranks and bilateral fusion categories

**Fig. 1:**
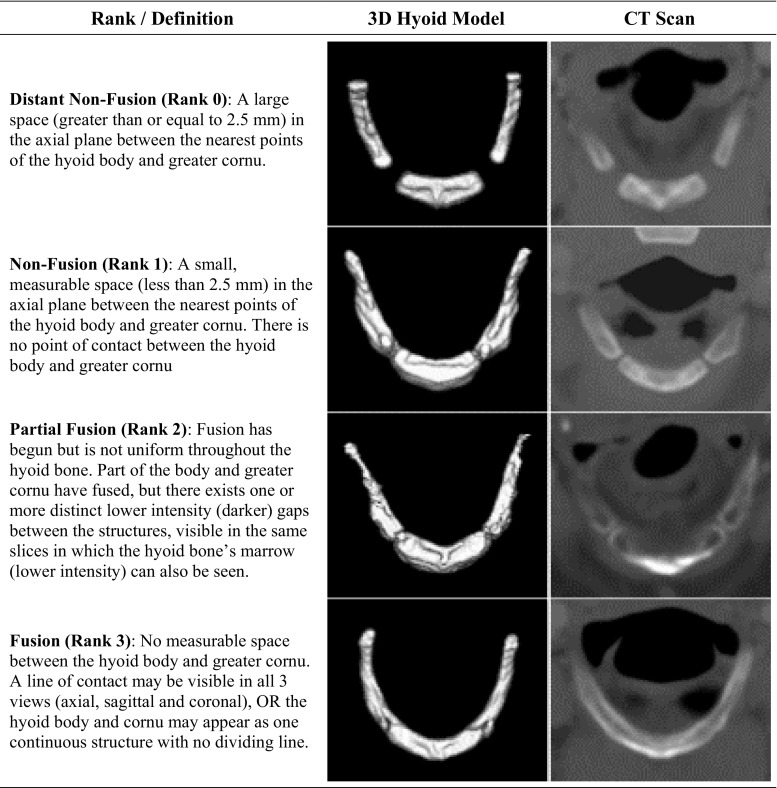
Fusion rank definition with images showing the same fusion rank bilaterally. The 3D Hyoid bone models were created using image visualization and analysis software Analyze 11.0^®^ (AnalyzeDirect^®^; Overland Park, KS). The axial slice from the CT scan with the smallest distance between the hyoid body and the greater cornua was used for fusion ranking but all axial, coronal, and sagittal views were inspected to accurately determine degree of fusion

For each hyoid bone, every axial CT slice was visually inspected in the Region of Interest tool in Analyze. The slice with the smallest distance between the hyoid body and the greater cornua on each side was used for fusion ranking using the following four ranks as defined in Fig. 1: 0. Distant non-fusion, 1. Non-fusion, 2. Partial-fusion, 3. Fusion. Fusion ranks were assigned to each side of the hyoid bone separately. Figure 1 also shows the 3D model and CT scan of the hyoid bones with each ranking. Hyoid bone fusion rankings entailed assessment of the hyoid bone in multiple planes bilaterally on CT images instead of the 3D hyoid models as it was often difficult to rank the degree of fusion present unless the CT slices were examined (see Fig. 1).

After examining the fusion ranks on the right and left sides (see Laterality in “Results” section below), we established the following four fusion categories: I. Bilateral distant non-fusion, II. Bilateral non-fusion, III. Partial or unilateral fusion, and IV. Bilateral fusion. Note that categories I, II, and IV have the same fusion ranks on both sides whereas category III includes a combination of fusion ranks where fusion has begun on at least one side but has not been completed bilaterally. This includes partial-fusion (rank 2) on one side with either non-fusion (rank 1), partial fusion (rank 2), or fusion (rank 3) on the opposite side; or fusion (rank 3) on one side and non-fusion (rank 1) on the opposite side. Table 1 describes the four bilateral categories of hyoid bone fusion.

**Table 1:** Fusion categories based on bilateral fusion ranks

Fusion categories	Definition	Image
I. Bilateral Distant Non-Fusion	Rank 0 (distant non-fusion) on both sides.	Fig. 1, row 1
II. Bilateral Non-Fusion	Rank 1 (non-fusion) on both sides.	Fig. 1, row 2
III. Partial or Unilateral Fusion	Rank 2 (partial fusion) on one side with: Rank 1 (non-fusion); Rank 2 (partial fusion); or Rank 3 (fusion) on the opposite side OR Rank 3 (fusion) on one side with Rank 1 (non-fusion) on the opposite side	Fig. 1, row 3 shows Rank 2 (partial fusion) on both sides. The 3 asymmetric types are not shown, but all occurred in our sample
IV. Bilateral Fusion	Rank 3 (fusion) on both sides	Fig. 1, row 4

Hyoid bone fusion was ranked by three raters. Fifteen scans were randomly selected for assessment of intra- and inter-rater reliability. Each rater ranked the right and left sides for a total of 30 rankings. Intra-rater agreement was 100 % for Rater 1. Inter-rater agreement among all three raters was 93 %. Weighted kappa (*κ*) for ordinal data with multiple raters [26] was used to test inter-rater agreement with *κ* = 0.906, 95 % CI 0.779–1.000.

### Data analysis

All analysis was completed using SAS© software, version 9.4 for Windows. An alpha level of 0.05 was used for significance in all statistical testing. Due to inconsistencies in previous findings on laterality and sex differences in fusion, we first examined the agreement of fusion ranks on the right and left sides, and then examined the association of sex and age to fusion rank on each side separately. Agreement of right and left sides was examined using Bhapkar’s test, which is appropriate for paired data with multiple categories [27, 28]. The association of fusion ranks with sex and age group were examined using Chi square tests of independence and Pearson correlation respectively. Based on the finding of no significant differences in laterality, we developed the four fusion categories using the bilateral fusion ranks described above and listed in Table 1. Sex and age analyses were repeated using the bilateral fusion categories rather than the fusion ranks for each side. A two-sample *t* test was used to examine sex differences in bone density in the pediatric age group. Two-way and three-way ANOVA models were used to investigate the association of sex, age, and fusion category with bone density in adults. Tukey’s honest significant difference (Tukey-HSD) was used for post hoc means comparisons. To examine whether bone density could contribute to age and sex estimation, we used both bone density and fusion category as predictors in generalized linear models. Two scans from adults in which density (mean intensity) could not be measured and one scan with an outlier value (>2.5 SD from the mean) were excluded from the density analysis and sex and age estimation models.

## Results

As detailed below, findings on hyoid bone fusion revealed no laterality in fusion, i.e., no significant differences in left versus right side fusion. Also, there was no relationship between fusion category and sex. Partial or complete fusion ranks were not present before age 20 years. While non-fusion decreased with age in all adults, the relationship of age group to fusion category differed by sex. As for hyoid bone density, findings for adults revealed females to have lower bone density than males and for density to decrease as age increased. Finally, there was no significant difference in bone density by fusion status.

### Fusion laterality

In the pediatric age group there was 100 % agreement of fusion ranks for right and left sides. Of the 46 hyoid bones, 41 were in category I, bilateral distant non-fusion and 5 were in category II, bilateral non-fusion. Therefore, the pediatric age group was excluded from further fusion laterality analysis. Table 2 shows the distribution of fusion ranks for the right and left side and the percentage agreement (i.e., the same rank on both sides) for males and females in the 3 adult age groups. The overall agreement was 74 %; agreement was slightly higher in females than males (79 vs. 70 % respectively), but tests of agreement were not significant for either sex (see Table 2). There was no significant association between sex and fusion rank on either side (left: *χ*^2^(2, *N* = 90) = 1.57, *p* = 0.455; right: *χ*^2^(2, *N* = 90) = 0.33, *p* = 0.847) and Pearson correlations between fusion ranks and age group were similar for both sides (left: *r* = 0.71, *p* < 0.001; right: *r* = 0.72, *p* < 0.001).

**Table 2:** Distribution and agreement of right and left fusion ranks for three adult age groups

Sex	Fusion rank	Left side		Right side		Agreement	Bhapkar’s test	
		*n*	*%*	*n*	%	%	χ^2^, *df* = 2	*p* value
Female	Non-fusion	22	47	21	45	79	0.52	.770
	Partial fusion	6	13	8	17			
	Fusion	19	40	18	38			
Male	Non-fusion	21	49	17	40	70	5.56	.062
	Partial fusion	9	21	9	21			
	Fusion	13	30	17	40			
Both	Non-fusion	43	48	38	42	74	2.10	.350
	Partial fusion	15	17	17	19			
	Fusion	32	36	35	39			

Based on the finding of no laterality in hyoid bone fusion, we established four fusion categories by combining the fusion ranks of the right and left sides as outlined in Table 1. All further analysis was completed using these categories. The number of individuals and percentage in each fusion category by sex and age group are shown in Table 3.

**Table 3:** Number of scans and percentage in each fusion category by sex and age group

Age group	Fusion category	Female		Male		Both	
		*n*	%	*n*	%	*n*	%
All ages	I. Bilateral distant non-fusion	19	27	22	33	41	30
	II. Bilateral non-fusion	23	33	15	23	38	28
	III. Partial or unilateral fusion	13	19	16	24	29	21
	IV. Bilateral fusion	15	21	13	20	28	21
Pediatric	I. Bilateral distant non-fusion	19	83	22	96	41	89
Birth-to-19 years	II. Bilateral non-fusion	4	17	1	4	5	11
Young adult	II. Bilateral non-fusion	12	80	7	47	19	63
20–30 years	III. Partial or unilateral fusion	2	13	3	20	5	17
	IV. Bilateral fusion	1	7	5	33	6	20
Middle-aged adult	II. Bilateral non-fusion	3	25	6	38	9	32
44–55 years	III. Partial or unilateral fusion	2	17	6	38	8	29
	IV. Bilateral fusion	7	58	4	25	11	39
Older adult	II. Bilateral non-fusion	4	20	1	8	5	16
75–95 years	III. Partial or unilateral fusion	9	45	7	59	16	50
	IV. Bilateral fusion	7	35	4	33	11	34

### Fusion: association with sex and age group

When age groups were combined, the distribution of fusion categories was similar for males versus females (see Table 3). There was no significant association between fusion category and sex with all age groups included (*χ*^2^ (3, *n* = 136) = 2.24, *p* = 0.524), or when restricted to adult age groups (*χ*^2^(2, *N* = 90) = 1.04, *p* = 0.596).

In the pediatric age group, 89 % of hyoid bones (100 % less than 14 years) were in category I, bilateral distant non-fusion, with the remaining 11 % in category II, bilateral non-fusion (see Table 3). Bilateral distant non-fusion did not occur past age 19 in our sample. In adults, there was a clear trend for category II, bilateral non-fusion, to decrease with age, with 63 % of young adults, 32 % of middle-aged adults, and 16 % of older adults in this category. This is reflected in the strong correlation between fusion category and age group (*r* = 0.75, *p* < 0.001) when all age groups were included, with similar correlations when stratified by sex (see Table 4). Partial or unilateral fusion increased with age for both sexes, with 17, 29, and 50 % of young, middle-aged, and older adults in this category respectively. For category IV, bilateral fusion, the trend was not strictly increasing for either sex. Middle-aged adult females had the highest percentage of bilateral fusion (58 %) as compared to 7 % for young adult females and 35 % for older adult females. In contrast, young adult males and older adult males had the highest percentages of bilateral fusion (33 % for both age groups) compared to 25 % for middle-aged adult males. These trends are reflected in a weaker but significant correlation of fusion with age in adult age groups (*r* = 0.31, *p* = 0.003). When stratified by sex, the correlation was significant for adult females (*r* = 0.42, *p* = 0.005), but not for adult males (*r* = 0.18, *p* = 0.284) (see Table 4). The fluctuating percentages of bilateral fusion were likely caused by sampling error as we do not know of any secular trend that would lead to decreasing fusion with age.

**Table 4:** Correlation of age group and fusion category for all age groups and three adult age groups, with and without stratification by sex

Age groups	Sex	Pearson *r*	95 % CI	*p* value
All	Female	.765	.670–.860	<.0001
	Male	.731	.618–.843	<.0001
	Both	.747	.671–.823	<.0001
Adults	Female	.424	.195–.652	.005
	Male	.183	−.100–.467	.284
	Both	.312	.125–.499	.003

### Bone density

**Fig. 2:**
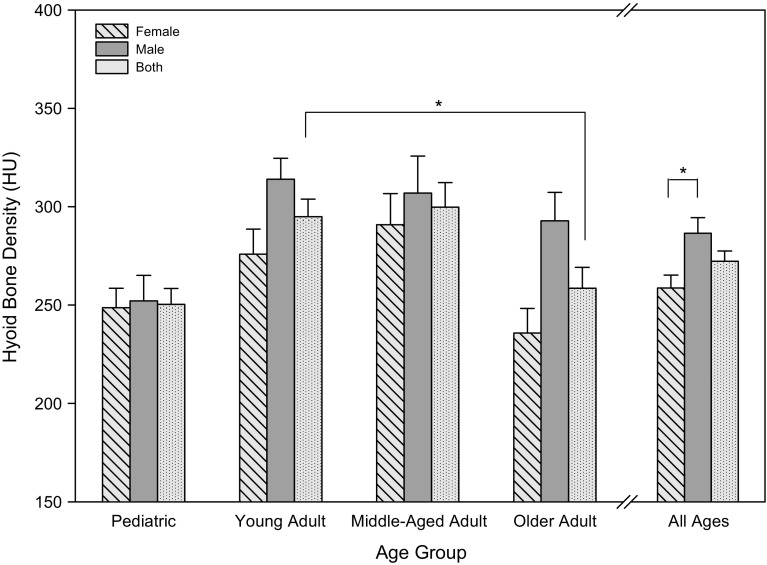
Hyoid bone density (mean intensity in HU) by sex and age group with standard *error bars*. *Lines* with *asterisks* (*) indicate significant post hoc differences (*p* < 0.05 Tukey-HSD) for sex and age in the 2- and 3-way ANOVA models. Sex and age groups include all fusion categories. The pediatric age group was not included in ANOVA models

Figure 2 shows hyoid bone density by age group for females, males, and both sexes combined. In the three adult age groups, females had lower bone density than males, with the largest difference occurring in older adults. In the pediatric age group, there was no significant difference between male and female hyoid bone density (*t*(44) = −0.22, *p* = .83). The pediatric age group was excluded from the 2-way and 3-way ANOVA models below because bone density increases throughout childhood and adolescence [29, 30] and because no partial fusion or fusion occurred in this age group.

Two-way ANOVA models were used to examine the relationship of hyoid bone density to age and sex in adult age groups. The main effects for sex (*F*(1, 82) = 9.15, *p* = .003) and age group (*F*(2, 82), *p* = .033) were significant. In post hoc means comparisons using Tukey-HSD, there was a significant difference in bone density between males and females [Least Squares (LS) means: Female = 266 HU, Male = 300 HU, *p* = .003] and between young adults and older adults (LS means: Young adult = 295 HU; Older adult = 262.04, *p* = .05). The Sex x Age group interaction was not significant and was excluded from the final model.

**Fig. 3:**
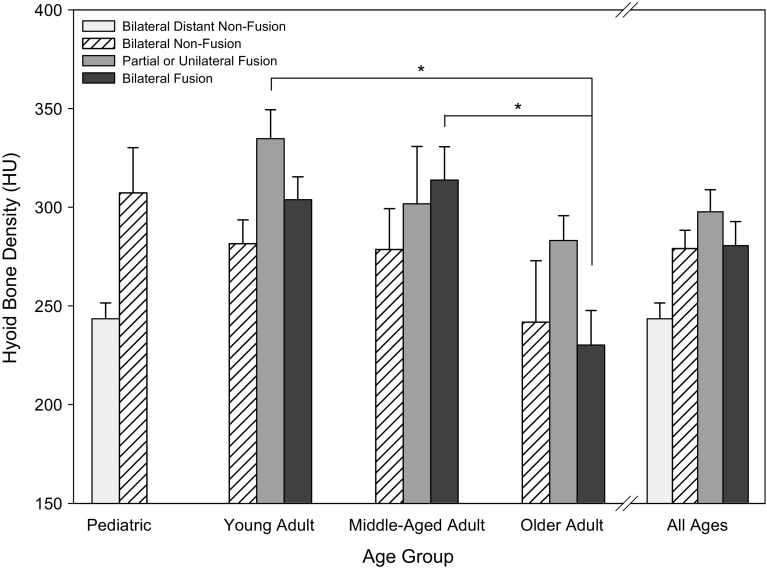
Hyoid bone density (mean intensity in HU) by fusion category and age group with standard *error bars*. *Lines* and *asterisks* (*) indicate significant post hoc differences (*p* < .05 Tukey-HSD) for the Age × Fusion interaction in the 3-way ANOVA model. Fusion category is based on fusion rank on both *right* and *left* sides (given no significant differences in laterality) and age and fusion groups include both sexes (given no significant Sex × Age × Fusion interaction). The pediatric age group was not included in the ANOVA model

Figure 3 shows hyoid bone density by fusion category and age group. The highest hyoid bone density occurred in young adults with partial or unilateral fusion while the lowest bone density occurred in adults with bilateral fusion.

Three-way ANOVA models were used to examine the association of bone density with fusion category, sex, and (adult) age group. As in the two-way ANOVA model, sex (*F*(1,76) = 9.86, *p* = .002) and age group (*F*(2,76) = 5.08, *p* = 0.009) were significant main effects. There were significant differences in density between males and females (Female = 265 HU, Male = 300 HU, *p* = .0024) and young adult and older adult age groups (Older adult = 257 HU, Young adult = 303 HU, *p* = .007) in post hoc comparisons. Fusion category was not a significant main effect (*F*(2,76) = 1.38, *p* = .26); however, the Age Group x Fusion Category interaction was significant (*F*(4,76) = 2.66, *p* = .039), indicating that the relationship between bone density and fusion category varied by age. In post hoc comparisons, young adults with partial or unilateral fusion (LS mean = 331.21 HU) and middle-aged adults with bilateral fusion (LS mean = 318.7 HU), had significantly greater bone density than older adults with bilateral fusion (233.74 HU; *p* values = .017 and .006 respectively; see Fig. 3). There were no significant differences in bone density by fusion category *within* age groups. All other 2- and 3-way interactions were not significant and were not included in the final model.

### Sex and age estimation models

In forensic science, there is an interest in estimating sex and age from the hyoid bone. Fusion status alone has been shown to be insufficient to estimate age [2, 12–14]. Dimensions of the hyoid bone, including the distance of the joint space between the greater cornua and hyoid body, have been used to predict sex and age in other studies [8, 9, 12, 31]. Given the association of bone density with sex and age group as well as the Age Group x Fusion Category interaction, we explored whether hyoid bone density could estimate sex and/or age.

Logistic regression models were used to estimate sex. Fusion category alone was not a significant predictor of sex, which confirmed the lack of association between fusion category and sex noted above. In contrast, hyoid bone density was a significant predictor: bone density less than 290.5 HU successfully classified 69 % of females, with 59 % of males with density greater than 290.5 HU (see Table 5). Fusion category was not significant when added to the density model.

**Table 5:** Results of generalized linear models^a^ using fusion category and bone density to estimate sex and age group: young adult, ages 20–30 years; middle-aged adult, ages 44–55 years; and older adult, ages 75–95 years

Response	Category	*n*	Fusion only				Fusion and/or Bone density^b^						
			%P	Fusion			%P	Fusion			Density		
				χ^2^	*df*	*p*		χ^2^	*df*	*p*	χ^2^	*df*	*p*
Sex	Female	45	73	1.30	2	.522	69	–	–	–	8.78	1	.003
	Male	41	37				59						
	Both	86	56				64						
F: Age group	20–30	15	80	11.98	4	.018	87	15.95	4	.003	10.17	1	.001
	44–55	12	58				50						
	75–95	18	44				83						
	All	45	60				76						
M: Age group	20–30	15	80	5.53	4	.237	73	5.24	4	.263	0.75	1	.386
	44–55	14	0				7						
	75–95	12	58				92						
	All	41	46				56						

*Fusion* fusion category (bilateral non-fusion, partial or unilateral fusion, bilateral fusion), *F* female, *M* male,  *%P* percent predicted correctly by model within a category, χ^2^ Wald Chi square, *df* degrees of freedom, *p* value

^a^Model type varied by response: logistic models were used for sex; cumulative logistic models for age group, with unequal coefficients for fusion category for each level of age

^b^Bone density alone was a significant predictor for sex. All other models included fusion category + bone density

Age group was estimated separately for males and females using cumulative logistic models. Fusion category was a significant predictor of age group for females, with a correct classification rate of 60 %. However, the associations did not reflect increasing fusion with age: non-fusion predicted young adults, partial or unilateral fusion predicted older adults, and bilateral fusion predicted middle-aged adults, which matched the most frequently occurring fusion category in each age group for females in our sample (see Tables 3, 5). When both density and fusion were included in the model for females, the overall correct prediction rate increased to 76 % (see Table 5). Higher bone density combined with either non-fusion or partial or unilateral fusion predicted young adults, lower bone density combined with any fusion category predicted older adults, and higher density with either partial or unilateral fusion or bilateral fusion predicted middle-aged adults. Details of the fusion categories and bone density ranges that predicted each age group for females are shown in Table 6. Neither fusion category nor bone density were significant predictors of age category for males (see Table 5).

**Table 6:** Fusion categories and bone density ranges that predicted each age group for females in the cumulative logistic model

Predictors		Age group predicted
Fusion category	Bone density (HU) range	
II. Bilateral non-fusion	204–368	Young females
III. Partial or unilateral fusion	357–368	
III. Partial or unilateral fusion	277–356	Middle-aged females
IV. Bilateral fusion	257–368	
II. Bilateral non-fusion	176–203	Older females
III. Partial or unilateral fusion	176–276	
IV. Bilateral fusion	176–256	

## Discussion

This study measured hyoid bone fusion and bone density from computed tomography scans and, to our knowledge, is the first study of its kind to examine developmental trends in hyoid bone density as well as the relationship between hyoid bone density and hyoid bone fusion. The results show no partial, unilateral, or bilateral fusion before age 20. Using four fusion categories, results show that all hyoid bones from cases under age 14 and half of 14–19 year olds showed bilateral distant non-fusion. No distant non-fusion was found over age 20. Bone density was higher in young adult and middle-aged adult groups and declined in older adulthood. Adult females had significantly lower hyoid bone density than adult males. Hyoid bone density successfully estimated sex in 64 % of the adult sample, and improved the estimation of adult female age group (from 60 to 76 %). There were no significant differences in bone density by fusion category within age groups. Our overarching goal is to contribute to the knowledge of vocal tract growth and development for the purpose of understanding speech and swallowing disorders, by way of an in vivo medical imaging database comprised of CT scans. This study contributes to the fields of forensics, speech pathology, and anatomy, by proposing a novel hyoid bone fusion ranking system that can be applied to other medical imaging databases and by demonstrating that hyoid bone density can be a useful addition to models estimating age and sex.

Our results are consistent with findings from previous research on the timing of fusion [2, 7–13]. We found no fusion before the age of 20, seven cases of complete fusion in the 20–30 year age group, and unfused hyoid bones in all age groups. We did observe sex differences in the age distribution of bilateral fusion, with more bilateral fusion in the young male adult and older male adult age groups, and more bilateral fusion in the middle-aged female adult group. However, this was likely due to a relatively small sample size and may not be representative of the general population. Our results revealed no differences in the fusion of the right versus left sides, a finding that is consistent with those of Parsons [7], Gupta et al. [8], Harjeet et al. [9] and Ito et al. [10] in which no differences were observed.

### Bone density

In adults, bone density was significantly lower in adult females than adult males, and lowest in older adult females. In the pediatric age group, there was no significant difference in male and female bone density.

To our knowledge, this study is unique in using hyoid bone density to estimate sex and age. Models to estimate sex using multiple hyoid bone measurements have been advanced [6, 12, 13, 31–33]. In these models, successful estimation of sex ranged from 69.2 to 89.5 % in males and 75.2–89.1 % in females. Ito et al. [10] used the volume of the hyoid bone, estimated from CT scans in an in vivo population, to estimate sex with 79 % success for female scans and 55 % success for male scans. In our study, bone density alone was a significant predictor of sex, with 69 % success for females and 59 % success for males. Bone density of the hyoid bone may be a useful addition to models that estimate sex from characteristics of the hyoid bone.

Several studies have noted that the joint space between the hyoid body and greater cornu decreases with age [9, 11, 12]. Naimo et al. [12], who evaluated 431 hyoid bones across the life-span, developed a regression model using joint space and hyoid bone measurements to estimate three broad age groups (pre-pubescent, 0–13 years; adolescent/young adult, 14–25 years; adult, 25+) with 87 % success. In our study, distant non-fusion was defined by distance >2.5 mm between the hyoid body and greater cornu. This category was a strong predictor of the pediatric age group (<20 years), with 89 % of the pediatric hyoid bones in this category (100 % <14 years, 50 % 14–19 years) and no hyoid bones from adults over age 20 in this category. The 2.5 mm distance may be an effective threshold for determining age < 20, especially in teens who may have hyoid bone dimensions that are similar to adults.

While the hyoid bone changes size and shape during development, Fakhry et al. [6] noted that the hyoid bone does not significantly change size or shape with aging past adolescence, which would preclude using measurement-based models to estimate age in adults. Findings from our sample of hyoid bones show that, as with other skeletal tissue, hyoid bone density decreased with age, with a greater decrease in females than males (see Fig. 2). Unlike males, middle aged adult females experience major hormonal variability around and after menopause, and bone health gradually declines with age [34]. Adding bone density to fusion category improved the estimation of age group in adult females from 60 to 76 %, with most of the improvement in prediction of the older age group (44–83 %; see Tables 5, 6). Considering bone density in addition to fusion status may be useful in distinguishing older adult females from young adult females.

Due to postmortem bone density loss and skeletal alteration, estimation of sex and/or age from hyoid bone density in a forensic setting may or may not retain the same percent success. Bell et al. [35] observed microstructural changes to bone as early as 3 months postmortem. Skeletal tissue demineralization is affected by, among other things, soil composition, vegetation cover, local climate, soil pH, as well as the type and growth of bacteria and fungi both in the environment and in the individual’s flora [35, 36]. Local burial customs and use of burial wrappings further contribute to the variation in rate of skeletal decomposition [36]. We could find no instances of comparison of bone density between in vivo and cadaver populations. Using hyoid bone density for sex or female age estimation would require further inquiries into how bone density changes during the postmortem interval.

### Implications for swallowing and speech disorders

The position of the hyoid bone relative to the other vocal tract structures changes during the course of development. Developmental changes, such as the descent of the hyo-laryngeal complex, are likely both structurally and functionally driven. The muscles and ligaments that are attached to the hyoid bone place pressure on it in several different planes. There are data to suggest that hyoid bones that remain unfused are better able to absorb the pressure that is put on them, making them less likely to deform with and change position relative to other vocal tract structures, such as the mandible and the pharynx, with increasing age [11]. Thus, fusion of the hyoid bone may affect its position in the vocal tract, which may have functional consequences. When the hyoid bone is positioned more inferior and distally to the mandible, for example, problems such as dysphagia, aspiration, and sleep apnea are more likely to occur [1, 37, 38]. The role that hyoid bone fusion plays in the functions of speech and swallowing has not been explored. Conversely, the role of extreme functions such as singing on hyoid bone fusion is unknown. Studies that explore the relational growth of the hyoid bone to structurally and functionally related structures such as the mandible and the larynx, as well as studies that assess the potential relationship between fusion and dysphagia are warranted.

### Limitations and future studies

In our cross-sectional study, bilateral fusion did not strictly increase with increasing age. Additional CT scans from both typically and atypically developing individuals, such as those with Down syndrome or cerebral palsy, would permit us to better understand the nature of hyoid bone fusion across the lifespan as well as the overall trends of hyoid bone form and function during growth and development. Cotter et al. [4] found that ex vivo hyoid bones taken from cadavers can be suitably compared to 3D renderings of in vivo hyoid bones created from CT scans. Additional measurements, including linear, angular and volume, of the hyoid bone from CT scans would permit us to test models that incorporate dimensions of the hyoid bone and bone density for understanding developmental trends and further refining sex and age estimation. Further investigation of bone mineral density changes in the postmortem interval could bridge the gap in knowledge on in vivo studies such as this and those of cadaver populations. Given our findings, which corroborate the work of Wan Harun [2], Naimo [12], D’Souza [14], and Miller [13], it is not recommended that fusion alone be considered a reliable method of age estimation in a forensic setting. The use of fusion as an age estimation tool should be applied only to those that show distant non-fusion. It may be possible to correctly identify an individual under the age of 20 years based on the distant non-fusion of his/her hyoid bone.

## Conclusion

The use of a clear fusion ranking system, such as the one used here can help clarify some of the inconsistencies in published research findings to date. Although increasing the number of retrospective imaging studies to boost the sample size used for this study would further confirm our conclusions, our findings indicate that a hyoid body/greater cornu joint space of >2.5 mm which was used to define distant non-fusion, was exclusively found in the 0–20 year age group. Bilateral non-fusion decreased with age, while partial or unilateral fusion increased with age. Broad categories of non-fusion versus fusion would not have captured these trends. As with other skeletal bone, hyoid bone density was lower in older adulthood, especially in females. Our results highlight the need to further explore the role of hyoid bone density as a useful addition to models that estimate age and/or sex. This research provides a baseline for understanding typical hyoid bone fusion and bone density development with possible implications for the fields of forensics, speech pathology, orofacial research, and anatomy.

## Key points

1. This study used the distance between the hyoid body and the greater cornua to establish a reliable hyoid bone fusion ranking system.
2. Fusion was ranked on each side, and hyoid bones (across the lifespan) were classified into one of four fusion categories based on their bilateral ranks: bilateral distant non-fusion, bilateral non-fusion, partial or unilateral fusion, and bilateral fusion.
3. There was no difference in the degree and timing of fusion for the right and left sides of the hyoid; also, there was no association of fusion category with sex when age groups were combined.
4. Despite a wide range of variability in the timing and degree of hyoid bone fusion, results showed that: distant non-fusion (>2.5 mm space between the hyoid body and greater cornua) only occured before age 20; after age 20 there was a trend for bilateral non-fusion to decrease with age into adulthood, but continued to be present in 16 % of the older adult (75–95 years) age group.
5. Bone density decreased with age for women. Including bone density with fusion status improved the prediction of age group for females, especially for the oldest age group. There was no association of fusion status or bone density with age group in males.
